# Augmented cerclage wire improves the fixation strength of a two-screw construct for humerus split type greater tuberosity fracture: a biomechanical study

**DOI:** 10.1186/s12891-021-04215-7

**Published:** 2021-04-12

**Authors:** Chao-Jui Chang, Wei-Ren Su, Kai-Lan Hsu, Chih-Kai Hong, Fa-Chuan Kuan, Chih-Hsun Chang, Cheng-Li Lin

**Affiliations:** 1 Department of Orthopaedic Surgery, National Cheng Kung University Hospital, College of Medicine, National Cheng Kung University, Tainan, Taiwan; 2 Skeleton Materials and Bio-compatibility Core Lab, Research Center of Clinical Medicine, National Cheng Kung University Hospital, College of Medicine, National Cheng Kung University, Tainan, Taiwan; 3 Medical Device Innovation Center (MDIC), National Cheng Kung University Hospital, Tainan, Taiwan; 4 Musculoskeletal Research Center, Innovation Headquarter, National Cheng Kung University, Tainan, Taiwan; 5 Institute of Biomedical Engineering, National Cheng Kung University, Tainan, Taiwan

**Keywords:** Humeral greater tuberosity fracture, Cerclage wire, Screw fixation, Biomechanics, Cadaver

## Abstract

**Background:**

Poor functional outcome can result from humeral greater tuberosity (GT) fracture if not treated appropriately. A two-screw construct is commonly used for the surgical treatment of such injury. However, loss of reduction is still a major concern after surgery. To improve the biomechanical strength of screw fixation in GT fractures, we made a simple modification of the two-screw construct by adding a cerclage wire to the two-screw construct. The purpose of this biomechanical study was to analyze the effect of this modification for the fixation of GT fractures.

**Materials and methods:**

Sixteen fresh-frozen human cadaveric shoulders were used in this study. The fracture models were arbitrarily assigned to one of two fixation methods. Group A (*n* = 8) was fixed with two threaded cancellous screws with washers. In group B (*n* = 8), all screws were set using methods identical to group A, with the addition of a cerclage wire. Horizontal traction was applied via a stainless steel cable fixed directly to the myotendinous junction of the supraspinatus muscle. Displacement of the fracture fixation under a pulling force of 100 N/200 N and loading force to construct failure were measured.

**Results:**

The mean displacements under 100 N and 200 N traction force were both significantly decreased in group B than in group A. (100 N: 1.06 ± 0.12 mm vs. 2.26 ± 0.24 mm, *p* < 0.001; 200 N: 2.21 ± 0.25 mm vs. 4.94 ± 0.30 mm, *p* < 0.001) Moreover, the failure load was significantly higher in group B compared with group A. (415 ± 52 N vs.335 ± 47 N, *p* = 0.01),

**Conclusions:**

The current biomechanical cadaveric study demonstrated that the two-screw fixation construct augmented with a cerclage wire has higher mechanical performance than the conventional two-screw configuration for the fixation of humeral GT fractures.

**Trial registration:**

Retrospectively registered.

## Introduction

Fractures of the proximal humerus account for 5% of all fractures, and greater tuberosity (GT) fractures are often part of this fracture [1, 2]. The GT is an important anatomic structure for shoulder abduction and external rotation, with isolated injury to this structure constituting 14 to 21% of all proximal humeral fractures [3–5]. Further, previous studies have shown that it is also involved in 13 to 33% of part of proximal humeral fractures [6–8]. Moreover, approximately 5 to 30% of anterior shoulder dislocations are complicated by a GT fracture [9–11]. Although isolated GT fractures may be subtle in their initial radiographic appearance, poor functional outcomes can result from these injuries if not treated appropriately [12, 13]. In the previous literature, surgical intervention is suggested for patients with more than 5 mm displacement of the GT fragment after fracture. Better functional and radiographic outcome could be anticipated when compared with nonoperative treatment [14]. The goal of surgical treatment is to restore the anatomic position of a displaced GT and to ensure the rotator cuff (RC) tendon integrity is preserved; in doing so, sufficient mechanical stability and early postoperative mobilization can be achieved [15].

In 2014, Mutch et al. proposed a morphologic classification of GT fracture, including avulsion, split and depression type, with the advantage to guide the technique of surgical treatment [16]. Split type fractures accounts for 40% of all GT fractures which represent the classic GT fracture as described in the Neer classification in which the fragment is large and the fracture line is parallel to the humeral shaft. The screw fixation is inexpensive and efficient and remains a cost-effective method to treat split type GT fracture [17–19].

Yet, this approach has the concern that the screw fixation of a GT fragment, especially in fragile bone, may be suboptimal in biomechanical strength. Thus, how to enhance the strength of screw fixations in GT fractures is an important and clinically relevant issue.

It has been demonstrated in a biomechanical study that the addition of a cerclage wire provides substantial improvement in mechanical performance regarding fixation of femoral neck fractures when compared with the conventional inverted triangle triple-screw construct [20]. In order to improve the biomechanical strength of screw fixation in GT fractures, we made a simple modification of the two-screw construct by adding a cerclage wire to the configuration. The purpose of this study was to analyze the biomechanical strength of these two different fixation constructs, using two screws alone or augmented with a cerclage wire, for the management of split type GT fractures. We hypothesized that the two-screw construct augmented with a cerclage wire would provide more biomechanical strength than the conventional two-screw construct.

## Materials and methods

### Specimen preparation

The institutional review board at our university hospital approved and evaluated this biomechanical cadaveric study. Sixteen fresh-frozen male human cadaveric shoulder specimens with an average age of 59.8 + 1.7 years were procured. Bone mineral density was determined before trials, which revealed non-osteoporotic bone quality (Table 1) [21]. Specimens, stored at − 20 °C prior to testing, were thawed for 24 h at room temperature in advance of dissection and experimentation. The skin, subcutaneous tissue, muscles, ligaments, tendons, scapula and clavicle were resected, with only the humerus and supraspinatus tendon retained. The supraspinatus tendon was preserved at the bony insertion over a length of 3 cm; meanwhile, the distal humeral condyle was detached, with a 20 cm humeral shaft length remaining.

**Table 1:** Bone mineral density and dimensions of the GT fragment

	Group A (***n*** = 8)	Group B (***n*** = 8)	***p*** value
**Bone mineral density (g/cm**^**2**^**)**	0.68 ± 0.007	0.69 ± 0.05	0.58
**Cross-sectional area (cm**^**2**^**)**	9.59 ± 0.86	9.49 ± 0.84	0.82
**Maximal thickness (mm)**	13.22 ± 1.01	13.08 ± 1.25	0.81

Standardized osteotomies oriented 50° to the humeral shaft were cut at the base of the GT using a thin blade reciprocating saw to create the split type GT fracture model. The osteotomy was initiated 2 mm medial to the footprint of the supraspinatus tendon and directly posterior to the bicipital groove [22–24]. The 16 fractured specimens were arbitrarily allocated to one of two fixation methods by a laboratory technician who was blind to the fixation construct, creating two groups with 8 specimens each. The same surgeon completed both fixation types for all specimens.

### Two-screw fixation constructs (Fig. 1)

**Fig. 1:**
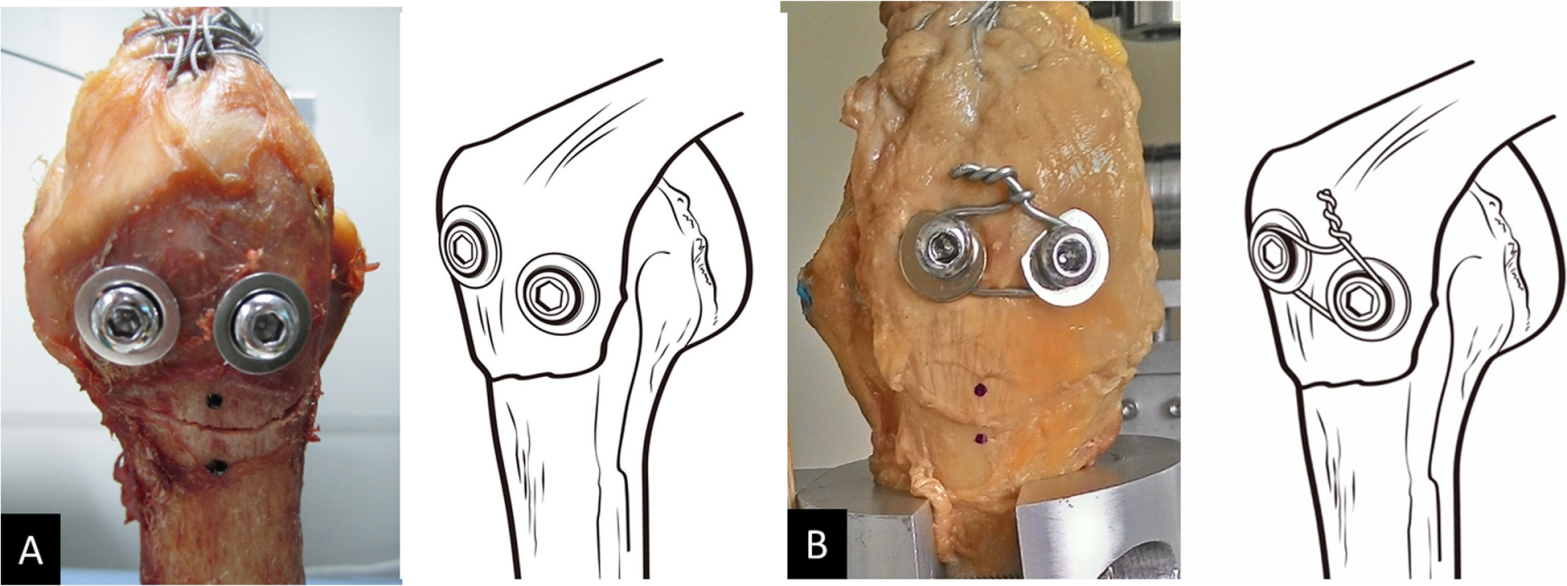
In group A (**a**), two threaded cancellous screws with washers were placed directly anterior and posterior to the central area of the fracture fragment. In group B (**b**), both screws were set using methods identical to group A, except for the addition of a cerclage wire

In group A (Fig. 1a), two threaded cancellous screws (diameter: 6.5 mm, Stryker) with washers were inserted just anterior and posterior to the central area of the fracture fragment. The distance between two screws was about 8-10 mm. The screw dimensions were chosen based on the length required to reach the opposite subcortical bone without embedding into the opposite cortex. Screw lengths ranged from 45 to 50 mm and there was no bone stripping on insertion.

Aside from the employed cerclage wire (No. 16, Aesculap) in group B (Fig. 1b), both screws were fixed with the identical approach as group A. The cancellous screws configured with washers were inserted 1-cm lateral to the cortex, thereby facilitating application of the cerclage wire. Following this, a No.16 wire was threaded in sequence through the gap between the screw and washer for both sets as the cerclage wire. Then, the screws were tightened so that the screw head and washer were flush with the lateral cortex of the GT. Lastly, the cerclage wire was tensioned to link the screws. Screw hole was drilled after the osteotomy and two screws were inserted in parallel fashion. The preparations of these two constructs were all performed by one experienced orthopaedic surgeon.

### Biomechanical testing

**Fig. 2:**
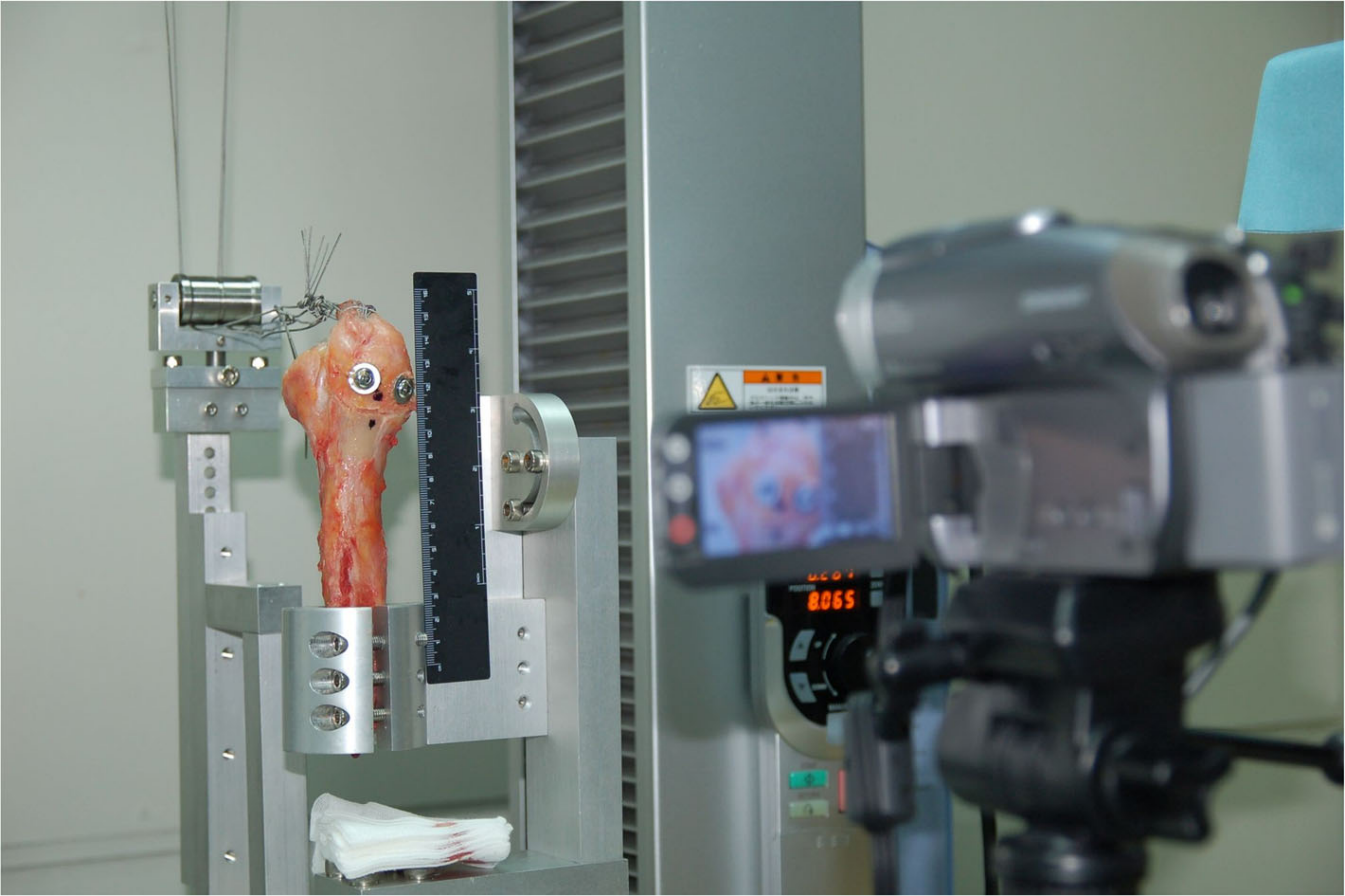
Mechanical testing setup

The humeral shaft was firmly secured to a custom-designed fitting mounted vertically onto the extended plate attached to the table of a universal material testing machine (MTS,AG-X, Shimadzu Corp., Tokyo, Japan). (Fig. 2) Horizontal traction was exerted with a stainless-steel cable attached to the myotendinous junction of each supraspinatus muscle using a Krackow technique. The cable was linked to the MTS via a pulley attached to the MTS table. The applied traction force was oriented at 0° of abduction [24]. A preload of 20 N was set, after which all constructs were subjected to 40 N of cyclical loading for 50 cycles, with a stepwise load increase of 20 N for 50 cycles until failure to simulate postoperative rehabilitation. A pulling speed of 60 mm/min was implemented. Ultimate failure was characterized as discontinuity in load–displacement curve or the maximum traction force at which a sharp decrease occurred.

Changes in displacement and the failure mode were recorded with a digital video camera (DCR-DVD 803, Sony Corp., Tokyo, Japan) for detailed assessment. Two indicators were marked on the GT and humerus distal to the GT to measure movement (Fig. 1). Movements between the indicators were measured using image analysis software (SigmaScan Pro 5.0, SPSS, Inc., Chicago, IL). Fixation strength was evaluated via three aspects: the movement under 100 N loading; the movement under 200 N loading; and the ultimate load to construct failure.

### Statistics

Statistical comparisons were performed with IBM SPSS Statistics for Mac. (Version 25; SPSS Inc.) To detect variations in fracture displacement under traction and failure load between the two groups, the Mann-Whitney U test was conducted. Significance was set at *p* < 0.05. A post hoc power analysis was carried out to confirm that enough specimens were included in each test group to identify all statistical differences to avoid type II error. The results showed that a sample size of at least 6 specimens per group would provide a study power of 0.8 and an α value of 0.05.

### Ethics approval and consent to participate

This cadaveric study was approved by The Institutional Review Board of National Cheng Kung University Hospital (approval number: ER-100-298). The institutional review board assessed and sanctioned this study. The fresh-frozen human cadaveric shoulder specimens were obtained from MedCure, Inc. Portland, USA. Consent for the storage and use of the bodies for research purposes was given by all body donors before death or by their next of kin. All methods were carried out in accordance with relevant guidelines and regulations.

## Results

There were no differences of bone mineral density between these two groups (*p* > 0.05). The GT fragments were also measured and showed no differences in cross-sectional area and maximal thickness between the two groups (*p* > 0.05). Mean and standard deviation for these measurements are outlined in Table 1.

**Fig. 3:**
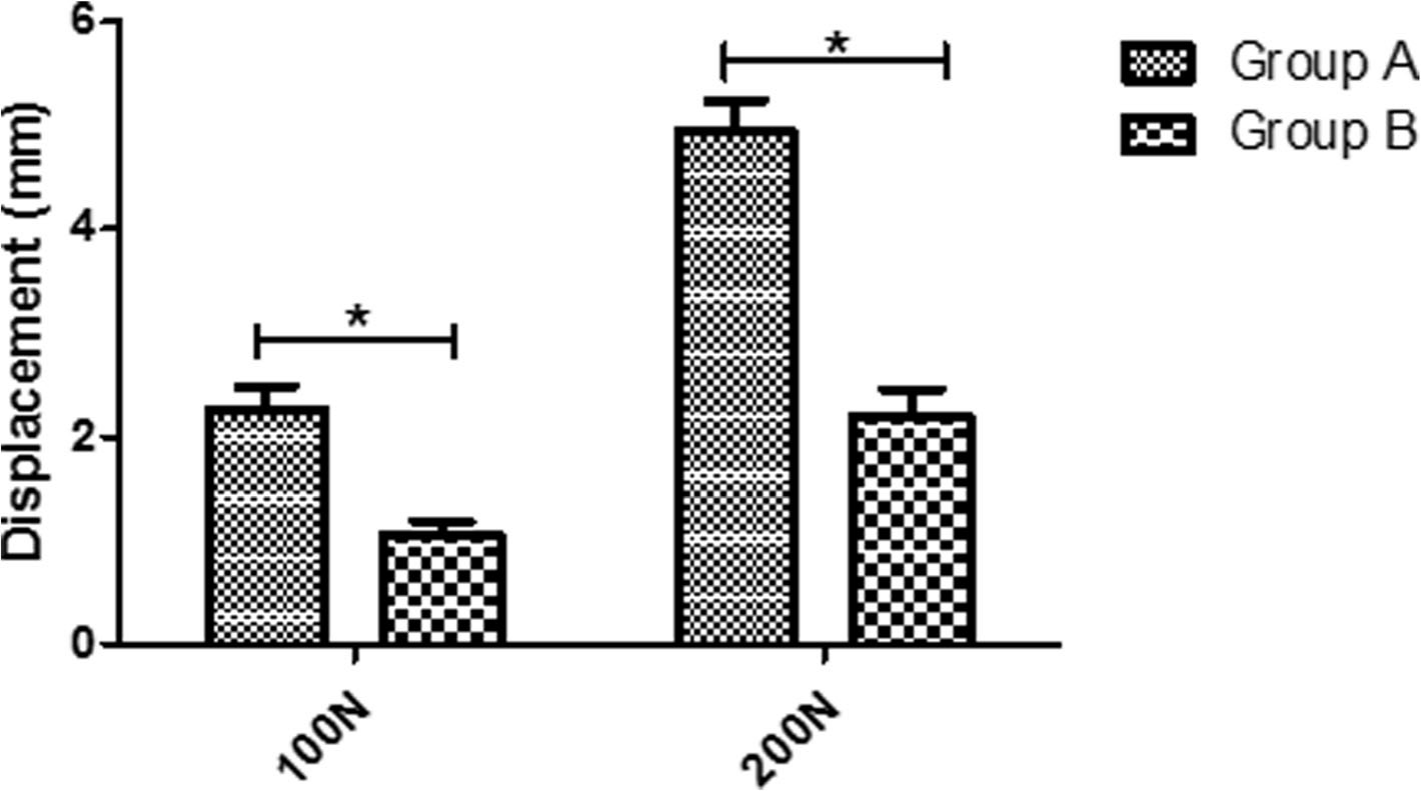
The mean displacement under a 100 N traction force was2.26 ± 0.24 mm for group A, and1.06 ± 0.12 mm for group B (*p* < 0.001). The mean displacement under a200 N traction force was4.94 ± 0.30 mm for group A, and2.21 ± 0.25 mm for group B (*p* < 0.001)

The mean displacement under the 100 N traction force was 2.26 ± 0.24 mm for group A, and 1.06 ± 0.12 mm for group B. (*p* < 0.001). The mean displacement under the 200 N traction force was 4.94 ± 0.30 mm for group A, and 2.21 ± 0.25 mm for group B (*p* < 0.001). Overall, there were significantly decreased displacements under both the 100 N and 200 N load for group B (Fig. 3).

**Fig. 4:**
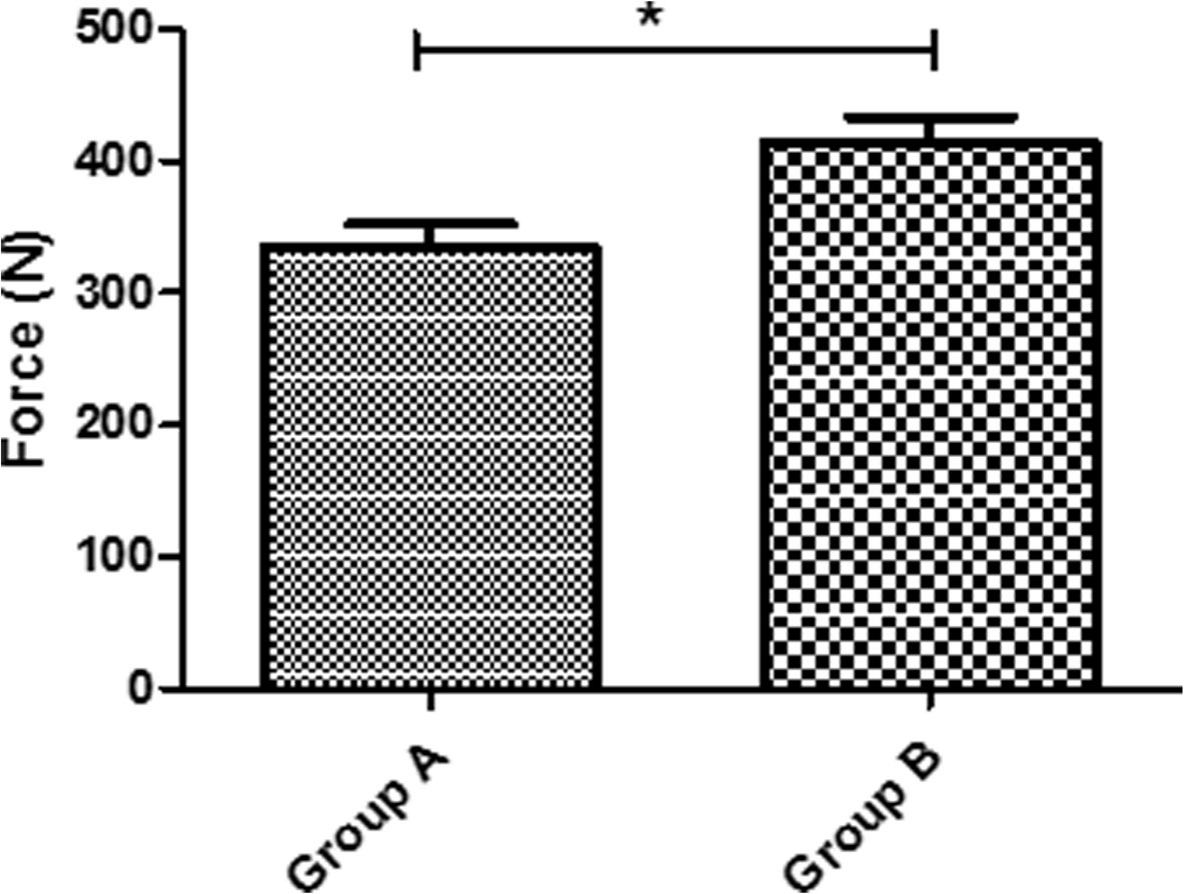
The mean traction force until load to failure was 335 ± 47 N for group A, and 415 ± 52 N for group B (*p* = 0.01)

Moreover, the mean traction force until load to failure was 335 ± 47 N for group A, and 415 ± 52 N for group B. Accordingly, there was also a significant difference between these two groups with respect to load to failure (*p* = 0.01) (Fig. 4).

### Mode of failure

All specimens in group A failed due to screw cut-out, leading to failure of the fixation construct itself. More than half of the specimen failures in group B occurred due to surgical neck fracture (5/8) rather than cut-out of the screws (3/8). Tendon attachment was intact in all specimens after failure of the fixation construct.

## Discussion

In the current biomechanical study, we compared the fixation strength of a conventional two-screw configuration to the same construct augmented with a cerclage wire for the treatment of humerus split type GT fracture. The result was in accordance with our hypothesis: the addition of a cerclage wire to the two-screw construct significantly decreased the fracture displacement in 100 N and 200 N traction forces, and also significantly increased the failure loading comparison to the conventional two-screw configuration.

Anatomic studies have shown that the GT is located 8 ± 3.2 mm inferior to the most superior aspect of the humeral head, lateral to the humeral head and posterolateral to the lesser tuberosity [25]. After GT fracture, the force vectors of the teres minor and the lower infraspinatus cause posterior displacement; meanwhile, the supraspinatus and upper infraspinatus result in superior displacement of the GT fragment [26]. When the displaced tuberosity is pulled posteriorly and superiorly, it may block the external rotation and abduction respectively [27, 28]. In addition, alteration of the rotator cuff attachment can lead to weakness of the rotator cuff and abnormal shoulder biomechanics. Previous biomechanical studies have indicated that 0.5 cm and 1.0 cm of superior displacement of the GT increases the necessary deltoid muscle force required for shoulder abduction by 16 and 27%, respectively, while a posterosuperior displacement of 1.0 cm increases the deltoid force by 29% [29]. Thus, Park et al. suggested that the fracture should be repaired if the displacement is more than 5 mm in young active patients, while fractures with 3 mm of displacement should be reduced in heavy laborers and athletes who are involved in overhead activity [30]. Our results showed that two-screw construct augmented with a cerclage wire had less than 3 mm displacement under 200 N load which was strong enough to bear rehabilitation interventions [31]. The two-screw construct augmented with a cerclage wire, average failure load more than 400 N, is expected to tolerate the maximal load from the supraspinatus of ~ 302 N [32].

40% of GT fractures are split fracture in which the fragment is large and the fracture line is parallel to the humeral shaft beginning proximally at the junction of the RC footprint and humeral head cartilage and extending distally and laterally to the level of the surgical neck [16]. Three methods of fixation have been described for split-type fractures: double-row suture-bridge, interfragmentary compression screws, or a small locking plate augmented with sutures through the RC tendon [17]. Suture anchor fixation have been described as an effective technique and a previous study, which compared the biomechanical strength of suture anchors and two-screw fixation in the management of split type GT fractures, showed that the suture anchor constructs were stronger than the fixation constructs using screws [24]. Nevertheless, the current results showed that the addition of a cerclage wire to the two-screw construct had the comparable biomechanical strength to the suture anchor constructs.

Open reduction with internal fixation (ORIF) and arthroscopic-assisted reduction with internal fixation (ARIF) can be employed to repair split-type fractures [33–35]. Some surgeons advocate plate and screw fixation for split GT fracture, and biomechanical study reveals that locking plate fixation provides the strongest and stiffest biomechanical fixation for split type greater tuberosity fractures [36, 37]. However, there remain concerns of more deltoid muscle dissection, axillary nerve injury [38], and subacromial impingement after osteosynthesis with plate and screws [39]. Previous studies have reported good functional recovery in treating isolated GT fractures with screw fixation [17–19]. A biomechanical analysis has also shown that strong fixation for isolated fractures of the greater tuberosity can be achieved by two cancellous screws [40]. Compared with plate fixation, there are advantages of screw fixation, including the less invasive approach, less intraoperative blood loss, lower risk for axillary nerve injury, and good shoulder function recovery [41]. Despite these advantages, screw fixation of a split GT fragment cannot always be performed due to insufficient bone stock, comminuted fracture, or osteoporotic bone. Moreover, screw fixation of a fragile GT fragment might lead to further comminution [42, 43]. Therefore, surgeons should exercise caution when using a screw fixation for GT fractures in older and osteoporotic bones.

French et al. suggested that the screws tightened by the wire loop provide a compressive force to counteract the varus force in supracondylar fractures of elbow [44]. Previous biomechanical analysis has also demonstrated that the addition of a cerclage wire provides substantial improvement in mechanical performance regarding fixation of femoral neck fractures when compared to the conventional inverted triangle triple screws construct [20]. The current results revealed that the two-screw construct augmented with cerclage wire significantly increased the biomechanical strength. This result may be attributed to the increased stability established by eliminating individual screw toggling. The washer played a role as the wire holder since the augmented wire was passed through the space between the screw and the washer. After screw insertion, the wire was twisted and tightened to connect the washers firmly. The augmented wire-screws construct was likely to serve as a single synthesized construct. Meanwhile, the stability of a femoral neck fracture was improved by adding an interlocking plate to three cannulated screws in the cadaveric biomechanical study [45]. The authors supposed that the synthesized construct comprising cerclage wire with cannulated screws had a similar function as the interlocking plate. More specifically, the entire construct reduced the motion between the screw and bone, and improved the strength of the femoral neck as well as GT fracture fixation.

**Fig. 5:**
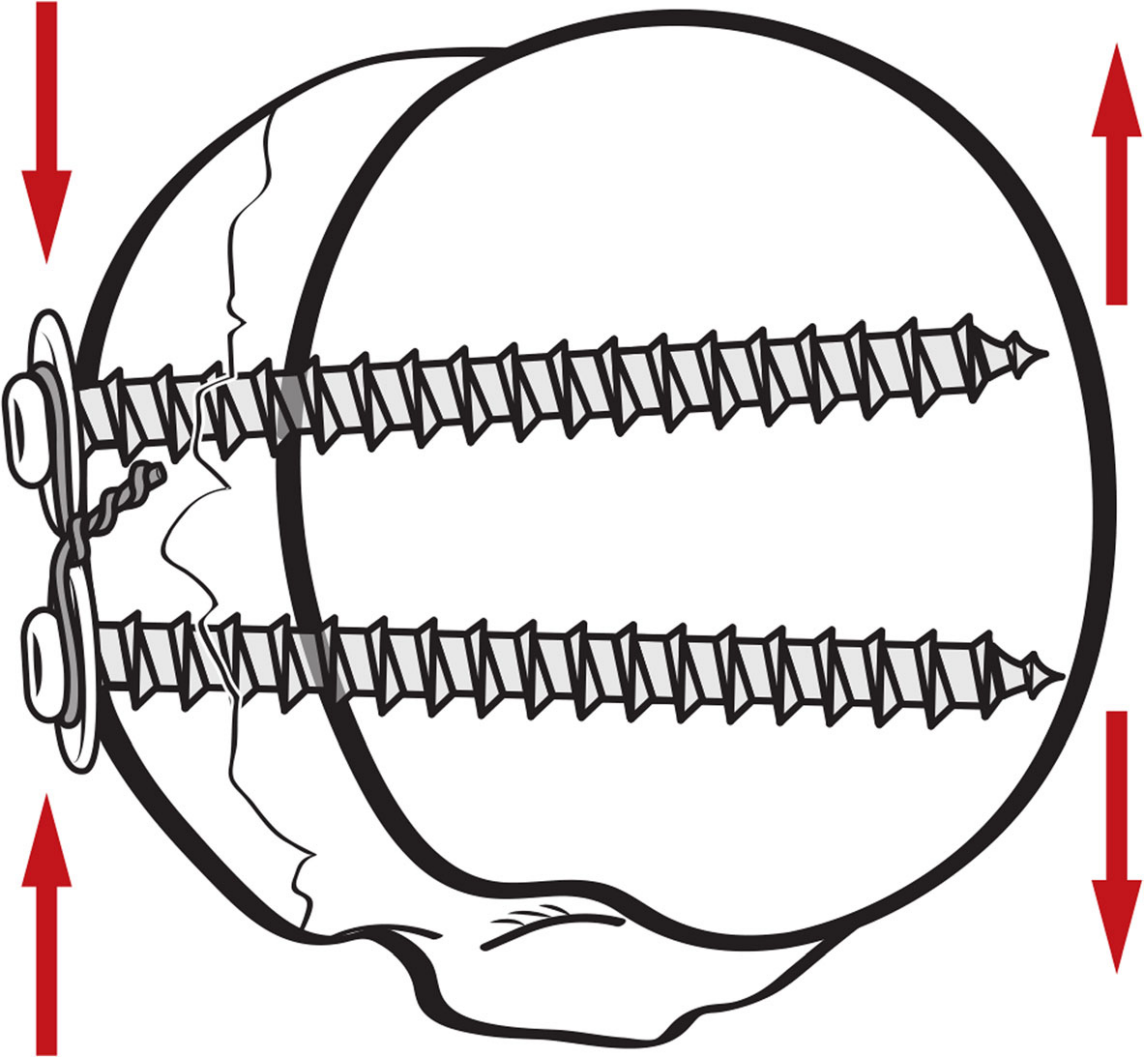
The two-screw construct augmented with a cerclage wire for the fixation of GT fracture. The distractive force produced by tightening of the augmented wire on the screws was directed against the subchondral bone of humeral head. Two screws were fixed in a divergent trajectories and perpendicular to the bone surface

Besides, tightening of the augmented wire might apply a distractive force on the screws, which provided the preload to the distracted screws. The force was directed against the subchondral bone which made the screw capture the humeral head firmly (Fig. 5). It could optimize the force to counteract the tension of the rotator cuff and reduce the micromotions. A previous study has proved that reduced micromotions might improve endosteal healing and sprouting angiogenesis [46]. These biological advantages may fasten the fracture healing in the early stage and theoretically decrease the risk of displacement, failure of the bone-implant construct, and mal-unions or non-unions. To our knowledge, this is the first study comparing the biomechanical strength of a screw-only construct and a screw-configuration construct augmented with a cerclage wire for the fixation of split type GT fractures. It provides support from a mechanical perspective for the treatment of isolated GT fracture of humerus.

This study has several limitations. Absolute standardization is difficult to realize because of the natural variations between cadaveric humeri regarding external and internal bone factors. Further, our results imply only the immediate postoperative strength because no healing can occur after the fixation. Fractures in the study were produced using a smooth saw, rather than the jagged features typically present at the interface between bone fragments in the clinical situation. Since actual forces on the humeral head are a combination of compression, torsion, and shear, the forces on the fixation construct may vary with different abduction angles. The loading pattern in this study was 0 degrees of abduction with a uniaxial direction. In addition, the model used here excludes some relevant forces, such as those of the infraspinatus and teres minor. These other forces may affect the clinical relevance of GT fractures. Therefore, our model, as with all models, may not represent the precise clinical situation. Last, although only one experienced orthopedic surgeon (CL Lin) performed all fixation constructs to maintain consistency, confounder might still exist. However, each of fixation construct in our study was completed manually to simulate clinical environment. Accordingly, future studies evaluating the strength of these constructs in different loading modes are needed.

In conclusion, this biomechanical cadaveric study demonstrated that a fixation construct augmented with a cerclage wire has superior biomechanical performance than a conventional two-screw configuration with respect to fixation of humerus split type GT fracture. It provides support from a mechanical perspective for the future clinical application of a cerclage wire.

## Data Availability

All data generated or analysed during this study are included in this published article.

## References

[1] Court-Brown CM, Caesar B (2006). Epidemiology of adult fractures: a review. Injury.

[2] Mauro CS (2011). Proximal humeral fractures. Curr Rev Musculoskelet Med.

[3] Court-Brown CM, Garg A, McQueen MM (2001). The epidemiology of proximal humeral fractures. Acta Orthop Scand.

[4] Depalma AF, Cautilli RA (1961). Fractures of the upper end of the humerus. Clin Orthop.

[5] Lind T, Kroner K, Jensen J (1989). The epidemiology of fractures of the proximal humerus. Arch Orthop Trauma Surg.

[6] Horak J, Nilsson BE (1975). Epidemiology of fracture of the upper end of the humerus. Clin Orthop Relat Res.

[7] Kristiansen B, Barfod G, Bredesen J, Erin-Madsen J, Grum B, Horsnaes MW, Aalberg JR (1987). Epidemiology of proximal humeral fractures. Acta Orthop Scand.

[8] Rose SH, Melton LJ, Morrey BF, Ilstrup DM, Riggs BL (1982). Epidemiologic features of humeral fractures. Clin Orthop Relat Res.

[9] Rowe CR (1956). Prognosis in dislocations of the shoulder. J Bone Joint Surg Am.

[10] Weaver JK (1987). Skiing-related injuries to the shoulder. Clin Orthop Relat Res.

[11] Kocher MS, Feagin JA (1996). Shoulder injuries during alpine skiing. Am J Sports Med.

[12] Ogawa K, Yoshida A, Ikegami H (2003). Isolated fractures of the greater tuberosity of the humerus: solutions to recognizing a frequently overlooked fracture. J Trauma.

[13] Gruson KI, Ruchelsman DE, Tejwani NC (2008). Isolated tuberosity fractures of the proximal humeral: current concepts. Injury.

[14] Platzer P, Thalhammer G, Oberleitner G, Kutscha-Lissberg F, Wieland T, Vecsei V, Gaebler C (2008). Displaced fractures of the greater tuberosity: a comparison of operative and nonoperative treatment. J Trauma.

[15] Kristiansen B, Angermann P, Larsen TK (1989). Functional results following fractures of the proximal humerus. A controlled clinical study comparing two periods of immobilization. Arch Orthop Trauma Surg.

[16] Mutch J, Laflamme GY, Hagemeister N, Cikes A, Rouleau DM (2014). A new morphological classification for greater tuberosity fractures of the proximal humerus: validation and clinical implications. Bone Joint J.

[17] Rouleau DM, Mutch J, Laflamme GY (2016). Surgical treatment of displaced greater tuberosity fractures of the Humerus. J Am Acad Orthop Surg.

[18] George MS (2007). Fractures of the greater tuberosity of the humerus. J Am Acad Orthop Surg.

[19] Bonsell S, Buford DA (2003). Arthroscopic reduction and internal fixation of a greater tuberosity fracture of the shoulder: a case report. J Shoulder Elb Surg.

[20] Kuan FC, Yeh ML, Hong CK, Chiang FL, Jou IM, Wang PH, Su WR (2016). Augmentation by cerclage wire improves fixation of vertical shear femoral neck fractures-a biomechanical analysis. Injury.

[21] Park JY, Kim MH (2004). Changes in bone mineral density of the proximal humerus in Koreans: suture anchor in rotator cuff repair. Orthopedics.

[22] Lin CL, Yeh ML, Su FC, Wang YC, Chiang CH, Hong CK, Su WR (2019). Different suture anchor fixation techniques affect contact properties in humeral greater tuberosity fracture: a biomechanical study. BMC Musculoskelet Disord.

[23] Lin CL, Su FC, Chang CH, Hong CK, Jou IM, Lin CJ, Su WR (2015). Effect of shoulder abduction on the fixation of humeral greater tuberosity fractures: a biomechanical study for three types of fixation constructs. J Shoulder Elb Surg.

[24] Lin CL, Hong CK, Jou IM, Lin CJ, Su FC, Su WR (2012). Suture anchor versus screw fixation for greater tuberosity fractures of the humerus--a biomechanical study. J Orthop Res.

[25] Iannotti JP, Gabriel JP, Schneck SL, Evans BG, Misra S (1992). The normal glenohumeral relationships. An anatomical study of one hundred and forty shoulders. J Bone Joint Surg Am.

[26] DeBottis D, Anavian J, Green A (2014). Surgical management of isolated greater tuberosity fractures of the proximal humerus. Orthop Clin North Am.

[27] Neer CS (1970). Displaced proximal humeral fractures. I. Classification and evaluation. J Bone Joint Surg Am.

[28] McLaughlin HL (1963). Dislocation of the shoulder with tuberosity fracture. Surg Clin North Am.

[29] Bono CM, Renard R, Levine RG, Levy AS (2001). Effect of displacement of fractures of the greater tuberosity on the mechanics of the shoulder. J Bone Joint Surg Br.

[30] Park TS, Choi IY, Kim YH, Park MR, Shon JH, Kim SI (1997). A new suggestion for the treatment of minimally displaced fractures of the greater tuberosity of the proximal humerus. Bull Hosp Jt Dis.

[31] Phillips D, Kosek P, Karduna A (2018). The contribution of the supraspinatus muscle at sub-maximal contractions. J Biomech.

[32] Burkhart SS (2000). A stepwise approach to arthroscopic rotator cuff repair based on biomechanical principles. Arthroscopy.

[33] Lee SU, Jeong C, Park IJ (2012). Arthroscopic fixation of displaced greater tuberosity fracture of the proximal humerus. Knee Surg Sports Traumatol Arthroscopy.

[34] Li R, Cai M, Tao K (2017). Arthroscopic reduction and fixation for displaced greater tuberosity fractures using the modified suture-bridge technique. Int Orthop.

[35] Hu C, Zhou K, Pan F, Zhai Q, Wen W, He X (2018). Application of pre-contoured anatomic locking plate for treatment of humerus split type greater tuberosity fractures: a prospective review of 68 cases with an average follow-up of 2.5 years. Injury.

[36] Gillespie RJ, Johnston PS, Gordon VA, Ward PJ, Getz CL (2015). Using plate Osteosynthesis to treat isolated greater tuberosity fractures. Am J Orthop.

[37] Gaudelli C, Menard J, Mutch J, Laflamme GY, Petit Y, Rouleau DM (2014). Locking plate fixation provides superior fixation of humerus split type greater tuberosity fractures than tension bands and double row suture bridges. Clin Biomech.

[38] Park J, Jeong SY (2014). Complications and outcomes of minimally invasive percutaneous plating for proximal humeral fractures. Clin Orthop Surg.

[39] Mouraria GG, Zoppi A, Kikuta FK, Moratelli L, Cruz MA, Etchebehere M (2019). Anterolateral approaches for proximal humeral Osteosynthesis: a systematic review. Acta Ortopedica Brasileira.

[40] Braunstein V, Wiedemann E, Plitz W, Muensterer OJ, Mutschler W, Hinterwimmer S (2007). Operative treatment of greater tuberosity fractures of the humerus--a biomechanical analysis. Clin Biomech.

[41] Cao L, Weng W, Song S, Li H, Su J (2013). Comparison of effectiveness between minimally invasive cannulated screw and open reduction and plate fixation in treatment of humeral greater tuberosity fracture. Zhongguo Xiu Fu Chong Jian Wai Ke Za Zhi.

[42] Williams GR, Wong KL (2000). Two-part and three-part fractures: open reduction and internal fixation versus closed reduction and percutaneous pinning. Orthop Clin North Am.

[43] Green A, Izzi J (2003). Isolated fractures of the greater tuberosity of the proximal humerus. J Shoulder Elb Surg.

[44] French PR (1959). Varus deformity of the elbow following supracondylar fractures of the humerus in children. Lancet.

[45] Basso T, Klaksvik J, Foss OA (2014). The effect of interlocking parallel screws in subcapital femoral-neck fracture fixation: a cadaver study. Clin Biomech.

[46] Basso T, Klaksvik J, Foss OA (2014). Locking plates and their effects on healing conditions and stress distribution: a femoral neck fracture study in cadavers. Clin Biomech.

